# Alpha-Synuclein and Calpains Disrupt SNARE-Mediated Synaptic Vesicle Fusion During Manganese Exposure in SH-SY5Y Cells

**DOI:** 10.3390/cells7120258

**Published:** 2018-12-08

**Authors:** Can Wang, Zhuo Ma, Dong-Ying Yan, Chang Liu, Yu Deng, Wei Liu, Zhao-Fa Xu, Bin Xu

**Affiliations:** Department of Environmental Health, School of Public Health, China Medical University, Shenyang North New Area, Shenyang 110122, Liaoning, China; wangcan_cmu@163.com (C.W.); mz_cmu@163.com (Z.M.); yanhan_cmu111@163.com (D.-Y.Y.); lc_cmu111@163.com (C.L.); dengyu.cmu@163.com (Y.D.); liuw@cmu.edu.cn (W.L.); zfxu@cmu.edu.cn (Z.-F.X.)

**Keywords:** manganese, SNARE complex, alpha-synuclein, synaptic vesicle fusion, neurotoxicity

## Abstract

Synaptic vesicle fusion is mediated by an assembly of soluble N-ethylmaleimide-sensitive fusion protein attachment protein receptors (SNAREs), composed of syntaxin 1, soluble NSF-attachment protein (SNAP)-25, and synaptobrevin-2/VAMP-2. Previous studies have suggested that over-exposure to manganese (Mn) could disrupt synaptic vesicle fusion by influencing SNARE complex formation, both in vitro and in vivo. However, the mechanisms underlying this effect remain unclear. Here we employed calpeptin, an inhibitor of calpains, along with a lentivirus vector containing alpha-synuclein (α-Syn) shRNA, to examine whether specific SNAP-25 cleavage and the over-expression of α-Syn disturbed the formation of the SNARE complex in SH-SY5Y cells. After cells were treated with Mn for 24 h, fragments of SNAP-25-N-terminal protein began to appear; however, this effect was reduced in the group of cells which were pre-treated with calpeptin. FM1-43-labeled synaptic vesicle fusion decreased with Mn treatment, which was consistent with the formation of SNARE complexes. The interaction of VAMP-2 and α-Syn increased significantly in normal cells in response to 100 μM Mn treatment, but decreased in LV-α-Syn shRNA cells treated with 100 μM Mn; similar results were observed in terms of the formation of SNARE complexes and FM1-43-labeled synaptic vesicle fusion. Our data suggested that Mn treatment could increase [Ca^2+^]_i_, leading to abnormally excessive calpains activity, which disrupted the SNARE complex by cleaving SNAP-25. Our data also provided convincing evidence that Mn could induce the over-expression of α-Syn; when combined with VAMP-2, α-Syn prevented VAMP-2 from joining the SNARE complex cycle.

## 1. Introduction

Manganese (Mn) can act as a neurotoxicant at high concentrations. Excessive exposure to Mn, typically aerosolized in certain industrial workplaces, is known to cause a movement disorder characterized by extrapyramidal motor dysfunction, including bradykinesia, akinetic rigidity, and dystonia [1]. Mn is also a component of an antiknock gasoline additive, known as methylcyclopentadienyl Mn tricarbonyl (MMT), and combustion results in the release of Mn phosphates into the ambient air. Excessive exposure to Mn in animal models has been shown to result in neurodegenerative disorders in multiple brain regions, including the basal nuclei, substantia nigra (SN), and globus pallidus (GP)—this condition is commonly referred to as manganism [2]. The actual mechanisms underlying these effects are however largely unknown, but usually lead to neurotoxicity, mitochondrial dysfunction, aberrant signal transduction, and oxidative stress [3]. Emerging evidence also indicates that chronic Mn exposure can disturb synaptic vesicle fusion, thus representing one of the most important cellular and molecular correlates of neurodegenerative diseases [1].

Soluble N-ethylmaleimide-sensitive fusion protein attachment protein receptors (SNAREs) play critical roles in mediating synaptic vesicle fusion, as well as most other fusion events within cells [4,5,6]. The synaptic SNARE complex consists of three components: v-SNAREs (VAMP-2/synaptobrevin) associate with vesicle membranes, while t-SNAREs (syntaxin 1 and soluble NSF-attachment protein (SNAP)-25) are found in target membranes [5]. In a vesicular fusion event, the t- and v-SNAREs assemble into a four-helix bundle, pulling the two membranes together to cause fusion. The complexes then disassemble and enable SNARE proteins to perform another round of fusion [7]. Aside from forming part of the SNARE complex, VAMP-2 is also a member of the VAMP-2-synaptophysin complex. The VAMP-2-synaptophysin complex provides a reserve pool for VAMP-2, which enables synaptic vesicles to adjust to an increased demand for synaptic efficiency [5,8]. A range of animal models have shown a reduction in neurotransmitter release upon the over-expression of alpha-synuclein (α-Syn) [9,10], suggesting that α-Syn may act as a regulator of neurotransmission, altering or disrupting the SNARE driven fusion of synaptic vesicles.

α-Syn is a highly charged 140-amino acid heat-stable protein that is soluble and natively “unfolded” [11]. α-Syn localizes to synaptic termini, and exists in equilibrium between the cytosol and the synaptic membrane [12]. Because α-Syn is localized to the presynaptic terminal, its physiological function has frequently been linked to both synaptic transmission and the SNARE complex, the core fusion machinery for vesicle fusion. However, increasing evidence suggests that the over-expression of α-Syn acts as a key regulator in the pathology of synaptic vesicle fusion [13]. For example, the over-expression of α-Syn was shown to cause the reduction of dopamine release by interfering with a step downstream of vesicle docking in exocytosis [14], or by inhibiting the reclustering of synaptic vesicles at the active zone after endocytosis [10]. In addition, α-Syn may represent an important protein in the development of neurodegeneration. The over-expression of α-Syn might disrupt synaptic function, resulting in cognitive disturbance [15]. Therefore, although it is highly likely that the over-expression of α-Syn might interfere with the mechanism underlying vesicular fusion, the molecular mechanism of this process is completely unknown.

Our previous research using primary cultured neurons confirmed that exposure to Mn could disturb the expression of SNARE complex-associated proteins, along with the interaction of these proteins [8]. A potential mechanism governing the proteolytic cleavage of SNAP-25 is calpains, a potential candidate protease. The calpains are calcium-dependent proteases, which cleave several cytosolic, membrane, or cytoskeleton-associated proteins [16]. Calpains are activated in a wide range of pathophysiological conditions characterized by dysregulation of neuronal calcium homeostasis, including stroke, epilepsy, traumatic brain injury, and neurodegenerative disorders. Even tiny changes in [Ca^2+^]_i_ within the physiological range can result in significant variations in calpains activity [17]. Following stimuli, such as Mn-treatment, the intracellular calcium concentration increases, leading to the activation of calpains. Activated calpains can cause proteolysis, which could alter the integrity, localization, or activity of endogenous proteins, and thus change substrate functions.

**Figure 1:**
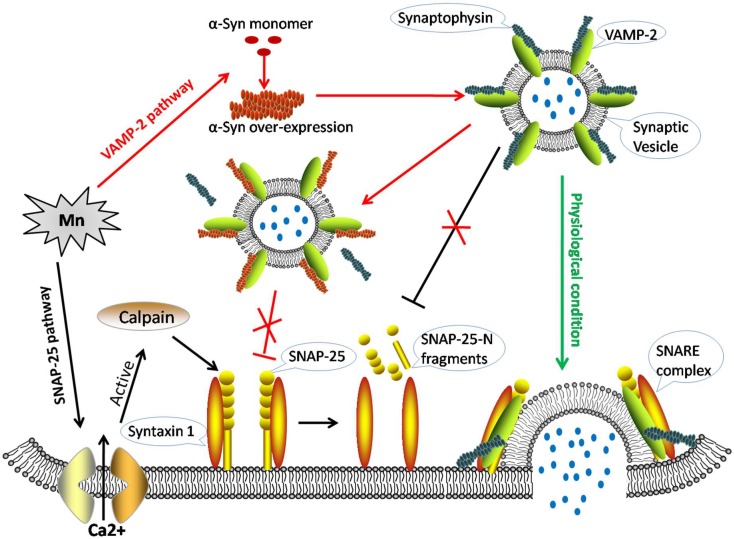
The molecular mechanisms of alpha-synuclein and calpains disrupting SNARE-mediated synaptic vesicle fusion during manganese exposure. Green arrow: In physiological conditions, synaptic vesicles are filled with neurotransmitters (blue dots). The VAMP-2-synaptophysin complex associates with the syntaxin 1-SNAP-25 complex, and the full soluble N-ethylmaleimide-sensitive fusion protein attachment protein receptor (SNARE)-complex assembly then pulls the membranes apart, opening the fusion pore to release the inner neurotransmitter. Black arrows: Mn increases [Ca^2+^]_i_ leading to the excessive activity of calpains, thus leading to the cleavage of soluble NSF-attachment protein (SNAP)-25. This fragmented form of SNAP-25 would not therefore be available for SNARE complex formation. Red arrows: Mn accelerates the over-expression of alpha-synuclein (α-Syn), which binds to VAMP-2 and blocks the combination of VAMP-2 with synaptophysin. Consequently, less VAMP-2 is available for SNARE complex formation.

Although we previously demonstrated that Mn could induce disorder of the SNARE complex assembly both in vitro and vivo [7,8], there is a paucity of data relating to whether α-Syn is involved in SNARE-mediated vesicle fusion. It is therefore critical that we evaluate Mn-induced disorders of synaptic vesicle fusion in a more extensive and systematic manner. We employed SH-SY5Y cell line for this research. SH-SY5Y cells are derived from -the human neuroblastoma cell line, while undifferentiated cells are more appropriate for studying neurotoxicity [18]. They could also be used to detect whether manganese is harmful to humans. In order to investigate the mechanism(s) underlying Mn induced SNARE-mediated synaptic vesicle fusion, we used calpeptin to inhibit the activity of calpains. We also created a SH-SY5Y cells model in which we used shRNA to knockdown α-Syn, and found that the abnormal activity of calpains and the over-expression of α-Syn were involved in Mn-induced synaptic vesicle fusion disorders (Figure 1).

## 2. Materials and Methods

### 2.1. Materials

SH-SY5Y neuroblastoma cells were purchased from American Type Culture Collection (ATCC, CRL-2266, Manassas, VA, USA). Manganese (II) chloride tetrahydrate (MnCl_2_·4H_2_O), DMEM and F-12, Fetal Bovine Serum (FBS), penicillin, streptomycin, and trypsin were supplied by Invitrogen (Carlsbad, CA, USA). Calpains, Calpeptin, SynaptoGreen^TM^C4 (FM1-43), mouse α-Syn, synaptophysin, and β-actin primary antibodies were purchased from Sigma (Saint Louis, MO, USA). PrimeScript^®^RT Enzyme Mix I and SYBR^®^Premix Ex TaqTMII kits were provided by TaKaRa Biotech. Co., Ltd. (Dalian, China). Rabbit SNAP-25-C-terminal and mouse SNAP-25-N-terminal primary antibodies were purchased from Synaptic Systems (Goettingen, Germany). Rabbit Syntaxin 1 and VAMP-2 primary antibodies, and horseradish peroxidase (HRP) conjugated anti-rabbit and anti-mouse secondary antibodies were supplied by Abcam Ltd. (Hong Kong, China). 

### 2.2. SH-SY5Y Cell Culture

SH-SY5Y cells were cultured in DMEM and F-12 supplemented with 10% FBS, 100 U/mL penicillin, and 100 g/mL streptomycin at 37 °C in a 5% CO_2_ humidified atmosphere. Medium was changed every 2 days and cells were cultured for 5–7 days. The cells were grown to 80% confluence for the next experiment.

### 2.3. Cell Treatment

We divided our experiment into three parts, each with a set of treatment groups. In the first part, cells were treated with 0, 50, 100, and 200 μM Mn in serum-free medium, and Cell Counting Kit-8 (CCK-8, Beyotime Biotech. Co. Ltd., cat no: C0038, Haimen, China) and lactate dehydrogenase (LDH) release assay was performed after cells were respectively treated for 0, 6, 12, 18, and 24 h. In subsequent experiments, Mn exposures were restricted for 24 h only, based on the results. In the second part, cells were treated with Mn (0 and 100 μM) for 24 h and calpeptin (1, 2, and 4 μM) dissolved in serum-free medium. In the third part, normal cells, and LV-α-Syn shRNA and LV-negative shRNA cells were respectively exposed to 0 and 100 μM Mn for 24 h in a serum-free medium.

### 2.4. Lentiviral Vector Construction and Transfection

For knockdown of human α-Syn (GenBank Accession no.6622), shRNA (5′-CGCGTCCCCGGAAGATATGCCTGTGGATCCTTCAAGAGAGGATCCACAGGCATATCTTCCTTTTTGGAAAT-3′) was designed as described previously [19]. pGCSIL-GFP-α-Syn shRNA, the α-Syn-RNA interference lentiviral vector, was constructed, and the recombinant virus was packaged using the lentivector expression system (Shanghai GeneChem Co., Ltd., Shanghai, China). A scrambled (Scr) shRNA was used as a negative control. To confirm the multiplicity of infection, cultured cells were co-transfected with the recombinant lentivirus for 72 h, until more than 90% green fluorescent protein in LV-α-Syn shRNA and LV-negative shRNA cells was observed using a fluorescence microscope. Verification of the silencing efficiency of genes was conducted using PCR and Western blotting technology. In the following sections, pGCSIL-α-Syn shRNA without GFP was used to protect the immunofluorescence experiment from interference. LV-α-Syn shRNA cells and LV-negative shRNA cells means the cells were transfected with α-Syn shRNA and Scr shRNA, respectively. The following assays were performed after confirming the best efficiency of transfection.

### 2.5. CCK-8 and LDH Release Assays

CCK-8 assay evaluates cell viability quantitatively. SH-SY5Y cells were seeded in 96-well plates (5 × 10^3^ cells/well) overnight. After treatment, cells were incubated with CCK-8 (20 μL per well) at 37 °C for 1h in accordance with the previously described method [20]. A microplate reader (Bio-Rad, Hercules, CA, USA) was used for measuring the values of absorbance at 450 nm. LDH release was measured by a method mentioned previously [21]. The microplate reader was used for measuring the values of absorbance at 490 nm. Both results of the absorbance of the treated wells were expressed as a percentage of the control wells.

### 2.6. Measurement of Intracellular Free Calcium

We assayed intracellular Ca^2+^ ([Ca^2+^]_i_) as described by He [22]. Cells were loaded with Fura-2 (20 µmol/L for 20 min) to quantify cytosolic [Ca^2+^]_i_, then a F-4500 Fluorescence Spectrophotometer (Hitachi, Tokyo, Japan) was used for calibrating absolute values from the measured fluorescence signals. Equation (1) was used for calibration:

[Ca^2+^]_i_ = K_d_ × [(R − R_min_)/(R_max_ − R)] × (S_f380_/S_b380_) (1)

where [Ca^2+^]_i_ is the concentration (nM) of intracellular Ca^2+^, K_d_ is the dissociation constant of the dye, R is the ratio at excitation wavelengths 340 and 380 nm, R_min_ is the ratio at zero [Ca^2+^]_I_, and R_max_ is the ratio at saturated [Ca^2+^]_i_ [23]. R_max_ and R_min_ were measured at the end of the experiments, because the procedures for obtaining them caused damage to cells. Firstly, R_max_ was obtained by adding 0.2% Triton X-100, making the cell membrane permeable to Ca^2+^ and allowing the extracellular Ca^2+^ and [Ca^2+^]_i_ to equilibrate. R_min_ was obtained next by adding the chelator EGTA 20 mM to chelate all Ca^2+^ inside and outside the cells. Cells were kept in Tyrode solution, in which pH was adjusted to 7.35 with NaOH at 37 °C. A K_d_ value of 224 nM was used. 

### 2.7. Measurement of Calpains Activity

The calpains activity was measured by a previously described method [24]. Briefly, after the cells were homogenized in an extraction medium containing 5 mM β-mercaptoethanol, 0.1 mM EDTA, lysocephalin 5 mM, DL-dithiothreitol 10 mM, and 20 mM Tris–HCl at pH 8.6, the homogenate was centrifuged at 1000× *g* for 10 min to remove the protein precipitate. For each sample, cell extract (containing 50 mg protein) was added to 96-well plates, and analysis of calpains activity was undertaken measuring the values of absorbance at 450 nm using the microplate reader. Calpains activity was expressed as fluorescent units. Based on the difference between samples with and without Ca^2+^, the calpains activity was calculated.

### 2.8. FM1-43 Fluorescence Image Analysis

As described previously, synaptic vesicle was marked by fluorescent dye FM1-43 and the release of it was measured by the decrease of FM1-43 fluorescence intensity [25]. Cells were incubated with FM1-43 (100 μM) for 2 min in hepes buffer medium (HBM) containing 1 mM Ca^2+^ and 30 mM K^+^, loaded with FM1-43 alone for 15 min. The fluorescent signal was tested first on an Olympus confocal microscope (FV 1000S-IX81, Olympus, Tokyo, Japan), using the 40× objective lens and a 488 nm argon laser. Next, cells were incubated with HBM containing 1 mM Ca^2+^ and 15 mM K^+^ for 30 min, the synaptic vesicles were then released, and the fluorescent signal examined again. The difference in relative fluorescence intensity before and 30 min after KCl-evoked exocytosis in different groups was normalized with those of control groups.

### 2.9. Quantitative Real-Time PCR Analysis

Total RNA was extracted from cells grown for 7 days in medium and using TRIzol (TaKaRa, Dalian, China) reagent according to the manufacturer’s instructions. Total RNA (1 μg) from each sample was reverse transcribed with the PrimeScript^®^ RT Enzyme Mix I (TaKaRa, Dalian, China) and oligo (dT) primers according to the manufacturer’s protocol. Real-time quantitative PCR (qPCR) was performed by the SYBR^®^ Premix Ex TaqTM II kit (TaKaRa, Dalian, China) using an ABI 7500 Real-Time PCR System (Applied Biosystems, CA, USA). Template cDNA (2 μg) was added to the final volume of 20 μL of reaction mixture. The PCR profile was: Denaturation at 95 °C for 30 s, and 40 cycles at each of 3 steps—95 °C for 30 s, 60 °C for 34 s, and 72 °C for 30 s. The primer sequence sets for α-Syn, syntaxin 1, SNAP-25, VAMP-2, synaptophysin, and β-actin are given in Table 1 [26,27]. The comparative CT method (ΔΔC_t_) was used for relative quantification of the genes tested.

### 2.10. Western Blotting

Cells were collected in Radio Immunoprecipitation Assay (RIPA) buffer containing phenylmethanesulfonyl fluoride (PMSF), a kind of common protease inhibitor. Protein concentrations were determined with the bicinchoninic acid (BCA) reagent from Pierce. Samples of total protein (20 μg) were separated by 12% polyacrylamide gel electrophoresis (120 V for 60 min) and transferred to polyvinylidene fluoride membranes (PVDF) (22 mm; Millipore, Ternicula, CA, USA) for 40 min at 100 A. PVDF membranes were blocked for 2 h at room temperature in TBST containing 5% dried skimmed milk, then the membranes were incubated with primary antibody in TBST overnight at 4 °C. The primary antibodies used were: α-Syn (1:500), syntaxin 1 (1:200), VAMP-2 (1:200), synaptophysin (1:200), β-actin (1:1000), SNAP-25-N-terminal (1:1000), and SNAP-25-C-terminal antibodies (1:1000). Treated membranes were incubated for 2 h at room temperature with HRP conjugated secondary antibodies (1:2000) after washing. Protein bands were detected using the Electro-Chemi-Luminescence (ECL) western blotting chemiluminescent detection reagents and autoradiography. Specific bands were analyzed semi-quantitatively by densitometry using image analyzing software (FluorChem v2.0, ProteinSimple, San Francisco, CA, USA). The changes of intensity of target proteins were normalized using the intensity obtained in the internal control bands (β-actin) [28].

### 2.11. SNARE Complex Analysis

Unboiled synaptic membrane proteins extraction was electrophoresed by western blotting to detect the SDS-resistant SNARE complexes [29]. Samples of total protein were separated by 4–20% gradient gels electrophoresis and transferred to PVDF membranes. After blocking, the PVDF membranes were incubated with syntaxin 1 antibody (1:1000), then with secondary antibody (1:2000), and bands were detected by ECL. All bands were normalized using the intensity obtained in the same sample (syntaxin 1 monomer level bands in normal western blotting).

### 2.12. Immunocytochemistry of VAMP-2 and α-Syn, VAMP-2, and Synaptophysin

After treatment, cells were fixed with 4% paraformaldehyde, then permeabilized and blocked in 0.1% Triton X-100. Next they were incubated in 5% donkey serum, then incubated with primary antibodies at 4 °C overnight. The primary antibodies were divided into two groups: One group was a mix of mouse anti-α-Syn antibody (1:100) and rabbit anti-VAMP-2 antibody (1:100), the other group was a mix of mouse anti-synaptophysin antibody (1:100) and rabbit anti-VAMP-2 antibody (1:100). Cells were incubated for 2 h at 37 °C with Alexa Fluor 488-labeled donkey anti-mouse IgG (1:1000) and Alexa Fluor 594-labeled donkey anti-rabbit IgG (1:1000). The fluorescent signal was examined on an Olympus confocal microscope (FV 1000S- IX81, Olympus, Tokyo, Japan), using the 40× objective lens. 

### 2.13. Co-Immunoprecipitation (Co-IP) of VAMP-2 and α-Syn, VAMP-2, and Synaptophysin

A Co-IP assay was performed as described previously [30]. After protein concentration normalization, the protein samples were incubated with rabbit anti-VAMP-2 polyclonal antibody (1:100), mouse anti-α-Syn monoclonal antibody (1:100), or mouse anti-synaptophysin (1:100) monoclonal antibody at 4 °C overnight to form an antigen-antibody complex. Then, immunomagnetic beads were pre-treated according to the manufacturer’s instruction and re-suspended in binding buffer (containing 50 mM Tris, 150 mM NaCl, 0.2% Triton-100; pH 7.5). The antigen-antibody complex solution was added into the binding buffer and incubated with beads for 2 h. The beads-antigen-antibody complex was collected by magnetic separation, loaded onto a 15% SDS-PAGE, and electro-transferred onto PVDF membrane. Membrane blots were probed with mouse anti-α-Syn, mouse anti-synaptophysin, or rabbit anti-VAMP-2 antibody, and visualized using a chemiluminescent detection system.

### 2.14. Statistical Analysis

All experiments were carried out at least three times. Results are expressed as mean ± SD. Differences between the means were determined by one-way ANOVA, followed by the Student–Newman–Keuls test, using SPSS 18.0 computer software (International Business Machines Corporation, New York, NY USA). *P* Values of less than 0.05 or 0.01 were considered significant.

## 3. Results

### 3.1. Mn Treatment Disturbed the Expression of Syntaxin 1, SNAP-25, and VAMP-2, and Reduced SNARE Complex Formation, in SH-SY5Y Cells

**Figure 2:**
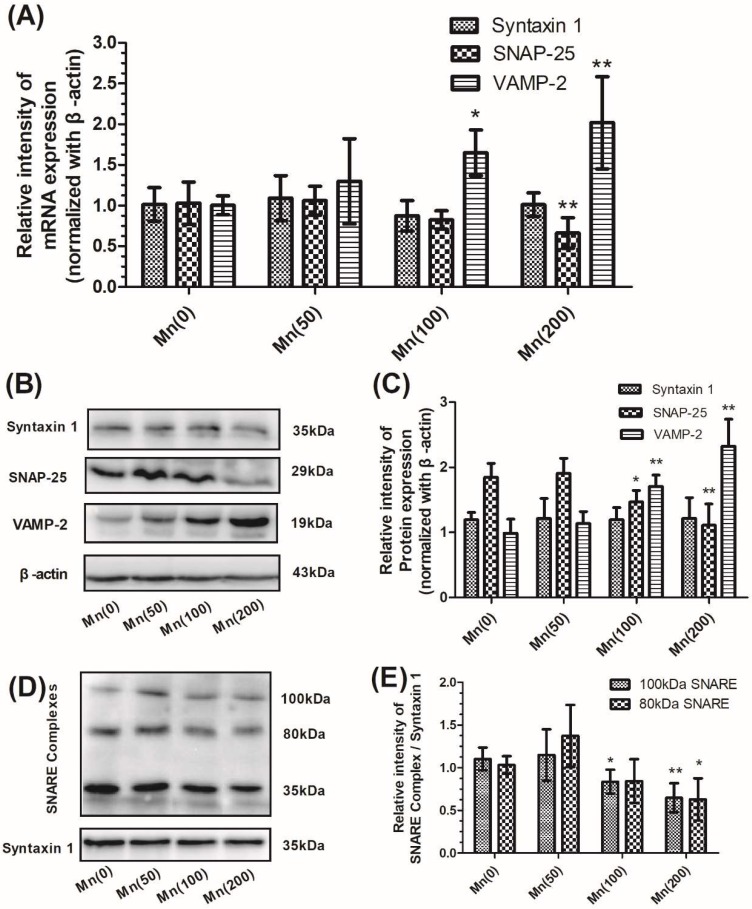
Mn disturbed the expression of syntaxin 1, SNAP-25, and VAMP-2, and reduced SNARE complex formation, in SH-SY5Y cells. After cells were exposed to 0, 50, 100, and 200 μM Mn for 24 h, the expression of syntaxin 1, SNAP-25, VAMP-2, and SNARE complexes were determined. (**A**) The mRNA expression of syntaxin 1, SNAP-25, and VAMP-2 normalized with β-actin. (**B**) The protein products of syntaxin 1, SNAP-25, VAMP-2, and β-actin. (**C**) Semi-quantitative analysis of the protein expression of syntaxin 1, SNAP-25, and VAMP-2 normalized with β-actin. (**D**) The protein products of the SNARE complex and syntaxin 1. Two major syntaxin 1-containing complexes were detected, migrating at 100 and 80 kDa, respectively. (**E**) Semi-quantitative analysis of the expression of the SNARE protein complex (100 and 80 kDa) normalized with syntaxin 1. Data represent mean ± standard deviation, *n* = 4. * *p* < 0.05 and ** *p* < 0.01 indicate significant differences from controls.

An optimized range of Mn concentrations (0, 50, 100, and 200 μM) was tested to determine the effect of Mn on cell viability (Figure S1). In this set of experiments, we quantified the total expression levels of cell mRNA and proteins forming the ternary SNARE complex, syntaxin 1, SNAP-25, and VAMP-2 by polymerase chain reaction (PCR) and western blotting. Exposure to Mn resulted in a significant increase of VAMP-2 mRNA expression in a concentration-dependent manner, with the maximum increase (2.01-fold relative to the control, *p* < 0.01) in cells treated with 200 μM of Mn; however, the expression of SNAP-25 mRNA decreased in a concentration-dependent manner (Figure 2A). As demonstrated in Figure 2B,C, exposure to Mn caused a significant increase of VAMP-2 protein expression, but resulted in a significant decrease by 39.95% of SNAP-25 protein expression in cells treated with 200 μM of Mn compared to controls. However, both the mRNA and protein expression of syntaxin 1 failed to show any significant changes in Mn-treated cells when compared to the controls (Figure 2A–C). Next, we determined the relative amounts of two major syntaxin-containing SNARE complexes, which migrated to molecular weights at 100 and 80 kDa, respectively (Figure 2D). As shown in Figure 2E, levels of both the 100 and 80 kDa complexes were significantly reduced (by 58.21% and 67.40% relative to control, *p* < 0.01 and *p* < 0.05, respectively) when cells were treated with 200 μM of Mn. Interestingly, the protein expression of SNAP-25 decreased significantly in cells treated with 100 μM of Mn, although the gene expression of SNAP-25 did change insignificantly. Based on this result, we suspected that there may be some forms of enzyme present which hydrolyze SNAP-25. In order to confirm our hypothesis, we selected the 100 μM Mn concentration to perform the following experiments.

### 3.2. Calpeptin Ameliorated SNAP-25 Cleavage and Promoted KCl-Evoked Synaptic Vesicle Fusion During Mn Exposure

To test our hypothesis we used calpeptin, an inhibitor of calpains, to explore the manner in which calpains was cleaved to SNAP-25. First we measured cell injury, [Ca^2+^]_i_, and the activity of calpains. As shown in Figure S2A, pretreatment with 4 μM calpeptin reduced Mn-induced cytotoxicity significantly; there was no obvious neurotoxicity in the 4 μM calpeptin treatment control group. As shown in Figure S2A, 100 μM of Mn significantly stimulated [Ca^2+^]_i_ and calpains activity (*p* < 0.01); calpeptin clearly inhibited the activity of calpains (*p* < 0.05), but had no effect on [Ca^2+^]_i_.

**Figure 3:**
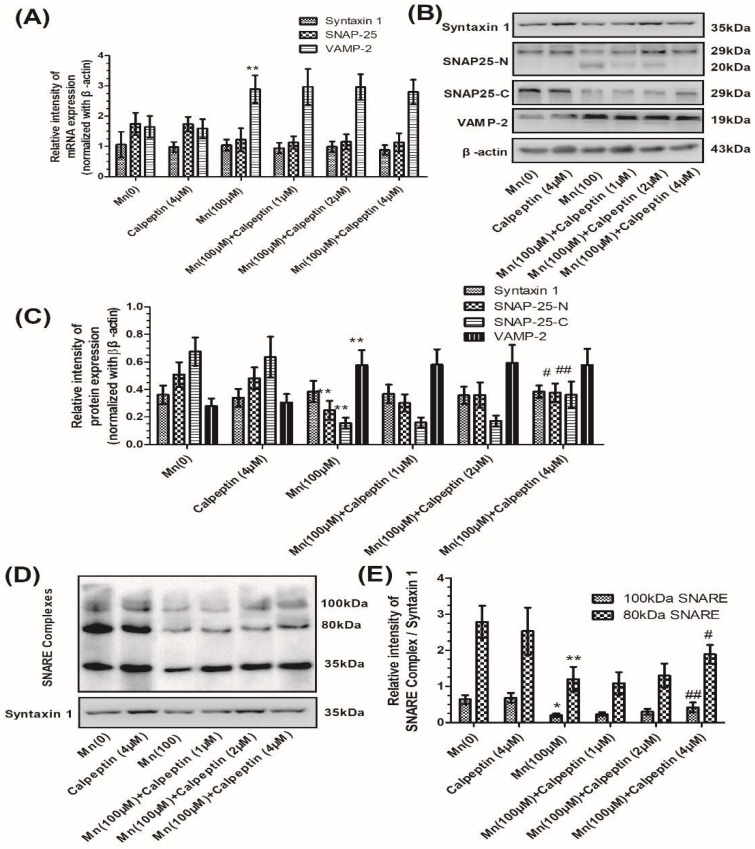
Calpeptin ameliorated SNAP-25 cleavage and promoted the formation of the SNARE complex. (**A**) The mRNA expression of syntaxin 1, SNAP-25, and VAMP-2 normalized with β-actin. (**B**) The protein products of syntaxin 1, N-terminal of SNAP-25, and C-terminal of SNAP-25, VAMP-2, and β-actin. (**C**) Semi-quantitative analysis of the protein expression of syntaxin 1, N-terminal of SNAP-25, and C-terminal of SNAP-25 and VAMP-2, normalized with β-actin. (**D**) The protein products of the SNARE complex. (**E**) Semi-quantitative analysis of the protein expression of SNARE complex protein normalized with syntaxin 1 protein. Data represent mean ± standard deviation, *n* = 4. * *p* < 0.05 and ** *p* < 0.01 indicate significant differences from controls; ^#^ *p* < 0.01 and ^##^ *p* < 0.01 indicate significant differences from the 100 μΜ Mn-treated group.

Next we examined the effect of calpeptin on the gene and protein expression of syntaxin 1, SNAP-25, and VAMP-2 during Mn exposure. Figure 3A shows that calpeptin did not change the level of mRNA expression for any of these three core proteins. As shown in Figure 3B,C, we determined SNAP-25 using two types of antibodies: A SNAP-25-N-terminus mouse antibody and a SNAP-25-C-terminus rabbit antibody. Both of these antibodies showed significant reductions of SNAP-25 in cells treated with 100 μM Mn, although levels increased when cells were pretreated with 4 μM calpeptin. In addition, pretreatment with 4 μM calpeptin caused an increase in the 80 kDa and 100 kDa protein forms of the SNARE complexes compared with cells treated with 100 μM of Mn (10.86- and 5.48-fold for the 100 μM Mn-treated group, *p* < 0.01 and *p* < 0.05, respectively) (Figure 3D, E).

**Figure 4:**
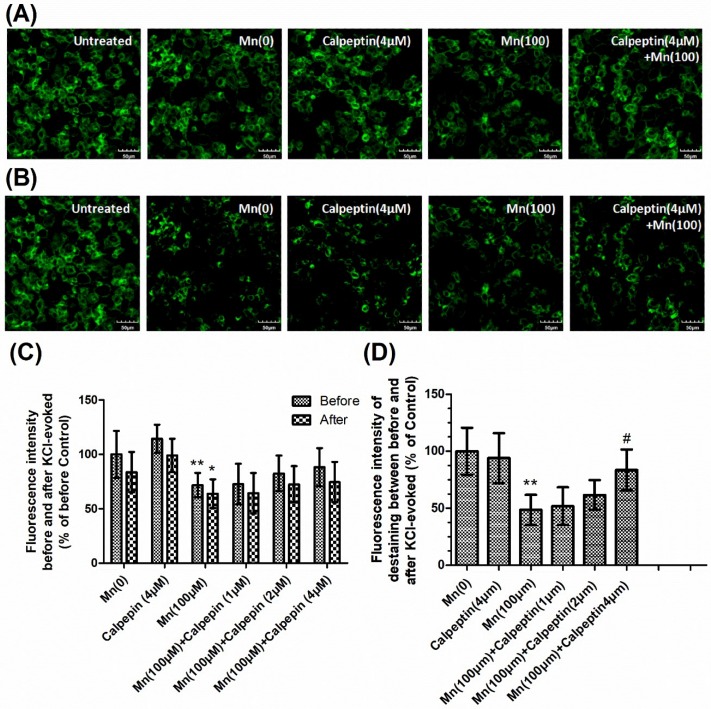
Calpeptin promoted KCl-evoked synaptic vesicle fusion. After treatment with different doses of Mn, cells were incubated with 100 μM FM1-43 for 2 min in HEPES buffer medium (HBM) containing 1 mM Ca^2+^ and 30 mM K^+^. (**A**) The FM1-43 fluorescence before KCl-evoked exocytosis. (**B**) The FM1-43 fluorescence 30 min after 15 mM KCl-evoked exocytosis. Untreated sample represented the normal downloading of the fluorescent dye in neurons neither KCl-evoked nor Mn-treated. (**C**) Fluorescence intensity before and after KCl-evoked exocytosis in different groups normalized with those of controls. (**D**) Relative fluorescence intensity between (**A**) and (**B**) in different groups normalized with those of controls. Data represent mean ± standard deviation, *n* = 4. * *p* < 0.05 and ** *p* < 0.01 indicates significant differences from controls; ^#^ *p* < 0.05 indicates significant differences from the 100 μΜ Mn-treated group. Scale bars: 50 μM.

In order to determine synaptic vesicle fusion, we employed FM1-43 fluorescence, a marker for firing neurons. The loss of FM1-43 fluorescence intensity was used to display the KCl-evoked exocytosis. Figure 4A shows FM1-43 fluorescence prior to KCl-evoked exocytosis, while Figure 4B shows fluorescence 30 min after KCl-evoked exocytosis. Untreated samples showed normal downloading of the fluorescent dye in the control group without KCl-treatment. We measured the fluorescence intensity before and after KCl-evoked exocytosis, and the relative fluorescence intensity between these, which had been normalized to the controls. Figure 4C shows that Mn reduced the loading of FM1-43 dye in cells, which means the number of active synaptic vesicles was decreased compared with control group. As shown in Figure 4C, the tendency was similar to the formation of SNARE complexes. Mn was shown to reduce the loss of FM1-43 fluorescence intensity, however calpeptin caused a significant increase in the relative fluorescence intensity of destaining compared to cells treated with 100 μM Mn. These results were consistent with the notion that calpeptin pretreatment can ameliorate Mn-induced synaptic vesicle fusion dysfunction.

### 3.3. α-Syn shRNA Promoted Interaction Between VAMP-2 and Synaptophysin, and Promoted KCl-Evoked Synaptic Vesicle Fusion During Mn Exposure.

According to the above experimental results, in the group of cells pretreated with 4 μM of calpeptin, the expression of full length SNAP-25 increased, while that of the N-terminal fragments disappeared; overall, SNARE complex protein formation was still lower than in the control group. We suspected that there was another factor present which could influence the formation of SNARE complex proteins. We used a lentiviral vector to knockdown α-Syn. Following transfection, knockdown efficiency was assayed by detecting the gene and protein expression of α-Syn in normal, LV-α-Syn shRNA, and LV-negative shRNA cells (Figure S3A,B). CCK-8 and LDH release assays were performed to test whether the lentivirus caused cellular damage. The resulting data showed that cells treated with LV-α-Syn shRNA and LV-negative-α-Syn had incurred no significant injury compared with the control group. The same results were obtained for CCK-8 (Figure S3C).

**Figure 5:**
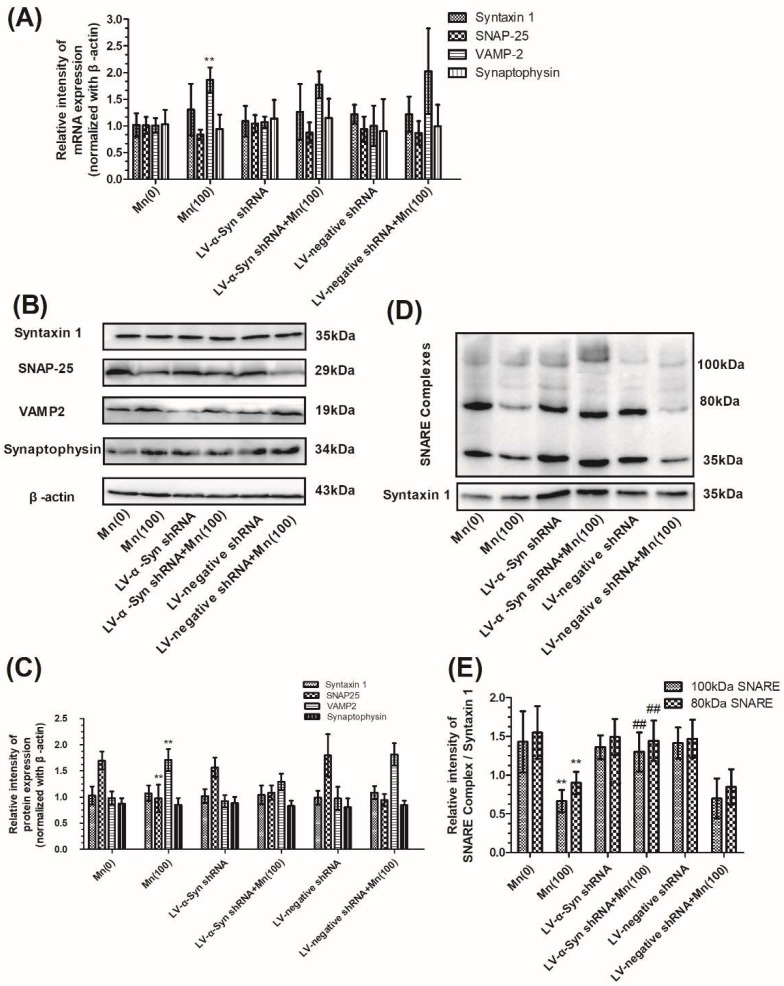
α-Syn shRNA did not change expression of VAMP-2 proteins, but promoted the formation of SNARE complex during Mn exposure. After normal and transfected cells were exposed to 100 μM of Mn, the mRNA and protein expression of syntaxin 1, SNAP-25, VAMP-2, and synaptophysin was determined. (**A**) The mRNA expression of syntaxin 1, SNAP-25, VAMP-2, and synaptophysin normalized with β-actin. (**B**) The protein products of syntaxin 1, SNAP-25, VAMP-2, synaptophysin, and β-actin. (**C**) Semi-quantitative analysis of the protein expression of syntaxin 1, SNAP-25, VAMP-2, and synaptophysin normalized with β-actin. (**D**) The protein products of the SNARE complex. (**E**) Semi-quantitative analysis of the protein expression of SNARE complex protein normalized with syntaxin 1. Data represent mean ± standard deviation, *n* = 4. ** *p* < 0.01 indicates significant differences from the controls; ^##^ *p* <0.01 indicates significant differences from the 100 μΜ Mn-treated group.

**Figure 6:**
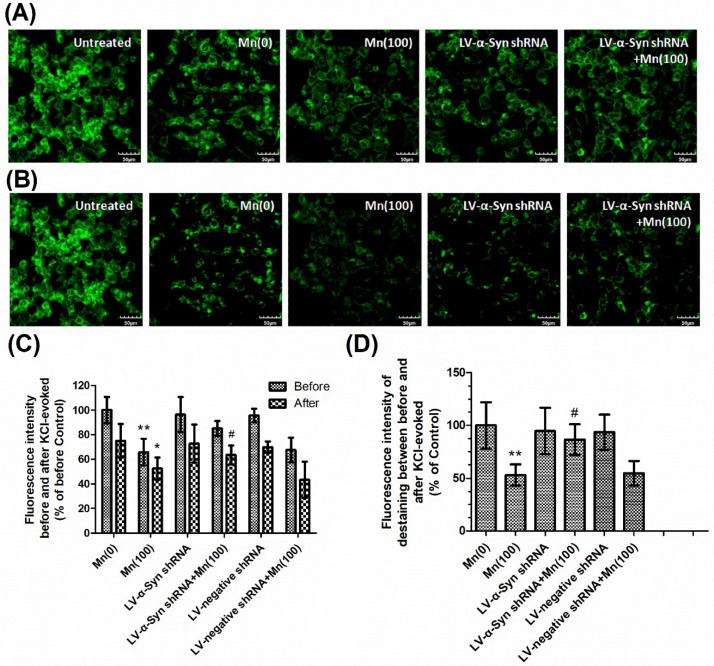
α-Syn shRNA promoted KCl-evoked synaptic vesicle fusion during Mn exposure. After treatment with different doses of Mn, cells were incubated with 100 μM FM1-43 for 2 min in HBM containing 1mM Ca^2+^ and 30 mM K^+^. (**A**) The FM1-43 fluorescence before KCl-evoked exocytosis. (**B**) The FM1-43 fluorescence 30 min after 15 mM KCl-evoked exocytosis. Untreated sample represented the normal downloading of the fluorescent dye in neurons neither KCl-evoked nor Mn-treated. (**C**) Fluorescence intensity before and after KCl-evoked exocytosis in different groups normalized with those of controls. (**D**) Relative fluorescence intensity between (**A**) and (**B**) in different groups normalized with those of controls. Data represent mean ± standard deviation, *n* = 4. * *p* < 0.05 and ** *p* < 0.01 indicates significant differences from controls; ^#^ *p* < 0.05 indicates significant differences from the 100 μΜ Mn-treated group. Scale bars: 50 μm.

Next, we examined the gene and protein expression of syntaxin 1, SNAP-25, VAMP-2, and synaptophysin in normal, LV-α-Syn shRNA, and LV-negative shRNA cells. As shown in Figure 5A–C, the lentiviral vector could not ameliorate changes occurring during Mn exposure. However, in the 100 μM Mn treatment group, the 100 and 80 kDa protein complex in LV-α-Syn shRNA cells showed significantly higher expression than normal cells (2.03- and 1.71-fold, respectively; *p* < 0.01; Figure 5D,E). Figure 6A–C shows the similar results using FM1-43 fluorescence to detect KCl-evoked exocytosis. This data was consistent with the notion that α-Syn over-expression disturbed SNARE-mediated synaptic vesicle fusion.

**Figure 7:**
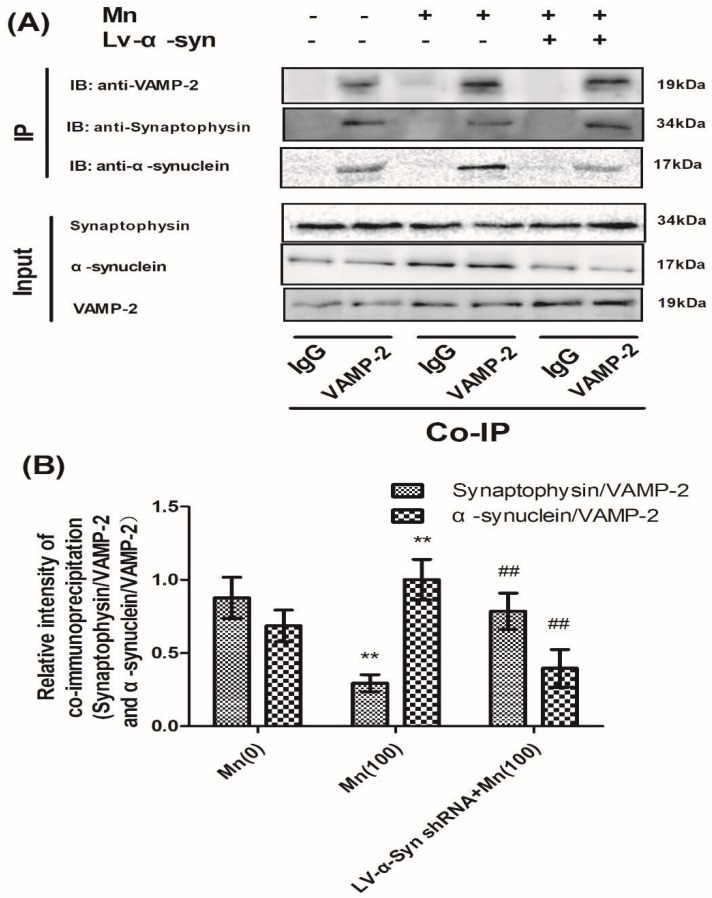
α-Syn shRNA reduced the protein interaction between VAMP-2 and α-Syn, but promoted the interaction between VAMP-2 and synaptophysin, during Mn exposure. (**A**) The immunoprecipitation products of VAMP-2 and α-Syn, and VAMP-2 and synaptophysin. (**B**) Semi-quantitative analysis of VAMP-2 and α-Syn, and VAMP-2 and synaptophysin. Expressions of synaptophysin and α-Syn were normalized with VAMP-2. Data represent mean ± SD, *n* = 4. ** *p* < 0.01 indicates significant differences from the controls; ^##^ *p* < 0.01 indicates significant differences from 100 μΜ Mn-treated group.

In order to further study the correlation between α-Syn and VAMP-2, we examined the interaction of VAMP-2 with α-Syn and VAMP-2 with synaptophysin in normal, LV-α-Syn shRNA, and LV-negative shRNA cells, using confocal laser scanning microscopy and co-immunoprecipitation. First, confocal microscopy was used to visualize the colocalization of these two groups of proteins in cells. Figure S4A shows that both VAMP-2 and α-Syn were present in cells, and that VAMP-2 co-localized with α-Syn in the overlay images. Similarly, Figure S4B shows that both VAMP-2 and synaptophysin were present in the cytoplasm and co-localized in the overlay images. Co-immunoprecipitation experiments demonstrated a direct association of these proteins in cells. Both proteins were identified in immunoprecipitates using specific antibodies (Figure 7A). As shown in Figure 7B, semi-quantitative analysis showed that Mn could promote interaction between VAMP-2 and α-Syn, but reduced the interaction between VAMP-2 and synaptophysin. As expected, a reduction in the expression of α-Syn could facilitate the correlation between VAMP-2 and synaptophysin.

## 4. Discussion

Mn induced disruption of neurotransmitter release may be a general consequence wherever Mn accumulates in the brain, and could underlie its pleiotropic effects [31]. Indeed, the SNARE complex and associated proteins are known to play a critical role in vesicle docking, priming, fusion, and synchronization of neurotransmitter release at presynaptic membranes. Previous work has established that the SNARE complex corresponds to the minimal machinery for membrane fusion in eukaryotic cells, forming a stable complex that renders the vesicles competent for fusion [32]. The highly-controlled interaction between vesicular and plasma membrane proteins is achieved by the formation of the ternary SNARE complex [33]. However, the mechanisms underlying Mn-induced SNARE-mediated synaptic vesicle fusion dysfunction are complicated and are not yet understood.

Our present study found that Mn exposure caused a concentration-dependent reduction in the expression of SNAP-25, and a concomitant increase in the expression of VAMP-2, in SH-SY5Y cells. We also found that the 80 kDa form of the SNARE complex first increased in abundance and then decreased. The 100 kDa form decreased in a concentration-dependent manner, which caused the SNARE complex assembly to become disordered. Similar data were reported in our previous study [7]. Interestingly, the protein expression of SNAP-25 decreased significantly in the 100 μM Mn-treated group compared with controls; however, the gene expression of SNAP-25 did not change significantly. Based on this result, we suspected there may be an as yet unidentified enzyme present, which could hydrolyze SNAP-25. In order to confirm our hypothesis, we selected a concentration of 100 μM Mn to perform the following experiments.

Previous research found that calpains are able to cleave various types of presynaptic proteins, including SNAP-25 [17]. Activated calpains are known to degrade a large number of cellular proteins, consequently calpains is considered to be an important mediator of vesicle fusion disorder [16]. The calpains cleavage site in SNAP-25 was expected to be situated at the C-terminal [17], therefore the full length SNAP-25 protein was tested using SNAP-25-C-terminal antibodies, while the N-terminal fragments were tested by SNAP-25-N-terminal antibodies. Previous studies indicated that Mn caused a significant increase in [Ca^2+^]_i_ by disrupting oxidative phosphorylation and increasing the generation of reactive oxygen species (ROS) [7,34], the overproduction of which might inhibit the Adenosine Triphosphate (ATP)-driven plasma membrane calcium-pump (Ca^2+^-ATPase). This pump is of crucial importance in maintaining a low resting [Ca^2+^]_i_ concentration [35]. Consequently, inhibition of this pump leads to changes in the regulation of Ca^2+^ levels [21]. Therefore, the inhibitory effect of Mn on Ca^2+^-ATPases was a potential factor underlying the significant increase in [Ca^2+^]_i_ in SH-SY5Y cells. In this study, treatment with 100 μM Mn resulted in a significant increase in the activity of calpains. We also showed that exposure to Mn caused the appearance of SNAP-25-N-terminal fragments, while expression of the full-length SNAP-25 protein decreased. It was also evident that calpain-mediated SNAP-25 fragmentation correlated with reductions in the SNARE complex.

In order to confirm the effect of calpains on the formation of the SNARE complex, we performed a series of experiments with calpeptin, an inhibitor of calpains. Our results indicated that calpeptin reduced the activity of calpains and partially promoted formation of the SNARE complex in SH-SY5Y cells. We also designed a calpeptin alone group to confirm that calpeptin is non-toxic, even at our highest dosages; results showed that there were no differences compared to the controls. Strikingly, in the group pretreated with 4 μM of calpeptin, expression of the full-length SNAP-25 protein increased significantly compared with the 100 μM Mn-treated group; the N-terminal fragments totally disappeared, which could have been due to the inhibition of calpains activity. Based on these data, we concluded that low doses of calpeptin can inhibit the pathological consequences of calpains activation, such as the cleavage of SNAP-25.

According to the above experimental results, in cells pretreated with 4 μM calpeptin, the expression of full-length SNAP-25 increased and the N-terminal fragments disappeared, however the formation of SNARE complex proteins was still lower than seen in the control group. We suspected that there was another factor present which was able to influence the formation of SNARE complex proteins. Transgenic mice, featuring the over-expression of human α-Syn, invariably exhibit signs of neurodegeneration. However, previous studies have also reported impairment in synaptic vesicle exocytosis and a reduction in neurotransmitter release using such models [10,36]. In our previous study, we found that Mn could induce α-Syn protein over-expression [37]. Subsequently, α-Syn bound to VAMP-2 in a preferential manner, thus resulting in inhibition of the docking between donor and acceptor vesicles. To further explore the mechanism responsible for how the over-expression of α-Syn disrupts assembly of the SNARE-complex, we used a lentivirus vector, featuring α-Syn shRNA, to transfect and knockdown the expression of α-Syn in SH-SY5Y cells. 

This study indicated that the exposure of α-Syn knockdown cells to 100 μM Mn resulted in a significant reduction in the level of α-Syn level compared with normal cells treated with 100 μM of Mn. Moreover, the interaction between α-Syn and VAMP-2 decreased, while the interaction between VAMP-2 and synaptophysin increased. However, knocking down the expression of α-Syn had no effect on the Mn-induced expression of SNARE complex-associated proteins. It is possible that the over-expression of α-Syn involves multiple binding sites for VAMP-2 on the v-vesicle, and that these additional binding sites sequester most of the VAMP-2 onto the vesicle, making less VAMP-2 available for SNARE complex formation. Alternatively, it is possible that multiple VAMP-2 binding sites, which become active upon the over-expression of α-Syn, bind VAMP-2 proteins from several vesicles to induce vesicle clustering, thereby limiting the number of v-vesicles available for docking. Moreover, LDH release and the viability of the 100 μM Mn-treated cells was also partially alleviated by knocking down the expression of α-Syn. This further confirmed that α-Syn over-expression caused damage to cells. Therefore, the over-expression of α-Syn protein cannot only injure cells but can also induce disorders of SNARE complex formation, thus causing neurodegenerative diseases [38].

The present study demonstrated that Mn could disrupt synaptic vesicle fusion by disordering SNARE complex formation. The underlying mechanism responsible for this might be that Mn increases [Ca^2+^]_i_, leading to excessive activity of calpains, thus disrupting the SNARE complex by cleaving SNAP-25. In addition, Mn treatment could also induce the over-expression of α-Syn, which combines with VAMP-2, thus preventing VAMP-2 from contributing to the SNARE complex cycle.

## Figures and Tables

**Table 1:** Primers used to establish the cDNA amplification standard curves by conventional PCR.

Gene	Oligonucleotide	Sequence
β-actin	Forward	5′-CCAACCGCGAGAAGATGA-3′
	Reverse	5′-CCAGAGGCGTACAGGGATAG-3′
α-Syn	Forward	5′-AAATGTTGGAGGAGCAGTGG-3′
	Reverse	5′-TCCAGAATTCCTTCCTGTGG-3’
Syntaxin 1	Forward	5′-TTTGCCCAGATGGTTCGACT-3′
	Reverse	5′-CAACCTTGACCTTGCCCATC-3′
SNAP-25	Forward	5′-CAGTTGGCTGATGAGTCGCT-3′
VAMP-2	Reverse	5′-TTCATGCCTTCTTCGACACGA-3′
	Forward	5′-CCAAGCTCAAGCGCAAAT-3′
	Reverse	5′-GGGATTTAAGTGCTGAAGTAAACTATG-3′
Synaptophysin	Forward	5′-CCAATCAGATGTAGTCTGGTCAGT-3′
	Reverse	5′-AGGGGTGGAGACCTAGGGTA-3′

## Supplementary Materials

The following are available online at http://www.mdpi.com/2073-4409/7/12/258/s1, Figure S1: Mn induced cell injury in SH-SY5Y. Figure S2: Calpeptin could ameliorate Mn-induced cell injury and the over-activation of calpains. Figure S3: α-Syn shRNA reduced α-Syn expression level and ameliorated cell injury during Mn exposure. Figure S4: Co-localization fluorescence signals of VAMP-2 and α-Syn, VAMP-2 and synaptophysin in cells.

## Abbreviations

α-Syn	Alpha-synuclein
Ca^2+^-ATPase	ATP-driven plasma membrane calcium-pump
CCK-8	Cell Counting Kit-8
GP	Globus pallidus
LDH	Lactate dehydrogenase
MMT	Methylcyclopentadienyl Mn tricarbonyl
Mn	Manganese
NSF	N-ethylmaleimide sensitive factor
qPCR	Real-time quantitative PCR
RNAi	RNA interference
ROS	Reactive Oxygen Specie
SN	Substantia nigra
SNAPs	Soluble NSF-attachment proteins
SNAREs	Soluble N-ethylmaleimide-sensitive fusion protein attachment protein receptors
