# Supplementary material for: Alpha-Synuclein and Calpains Disrupt SNARE-Mediated Synaptic Vesicle Fusion During Manganese Exposure in SH-SY5Y Cells
Source: Cells. 2018 Dec 8;7(12):258. doi: 10.3390/cells7120258 (PMC6316740; doi:10.3390/cells7120258)
Supplement: Supplementary file 1 [file cells-07-00258-s001.pdf]

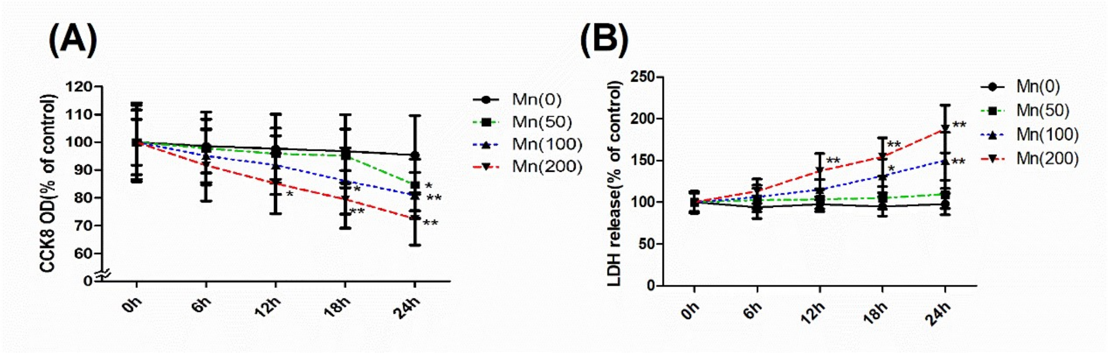


**Figure S1.** Mn induced cell injury in SH-SY5Y. In order to estimate the toxic effect of Mn, we measured the LDH released into the culture medium and used CCK-8 to evaluate the cell viability, after the cells had been treated with 0, 50, 100, 200 μM Mn for 0, 6, 12, 18, 24 h, respectively, cell viability was measured. (**A**) CCK-8 assay: after 200 μM Mn exposure for 24 h, the viability of cells was decreased by 27.51% significantly ($p < 0.01$). (**B**) LDH release assay: after cells were exposed to 200 μM Mn for 12, 18 and 24 h treatment period, the results showed significantly increased LDH levels in the culture medium (1.37-, 1.54- and 1.87-fold of control, respectively, $p < 0.01$) indicative of overt cytotoxicity. Data were mean ± S.D, n = 6, *$p < 0.05$ and **$p < 0.01$ significantly different from the controls. However, cells treated with 200 μM Mn within 24 h could induce significant cell damages but cell viability was above 50%.

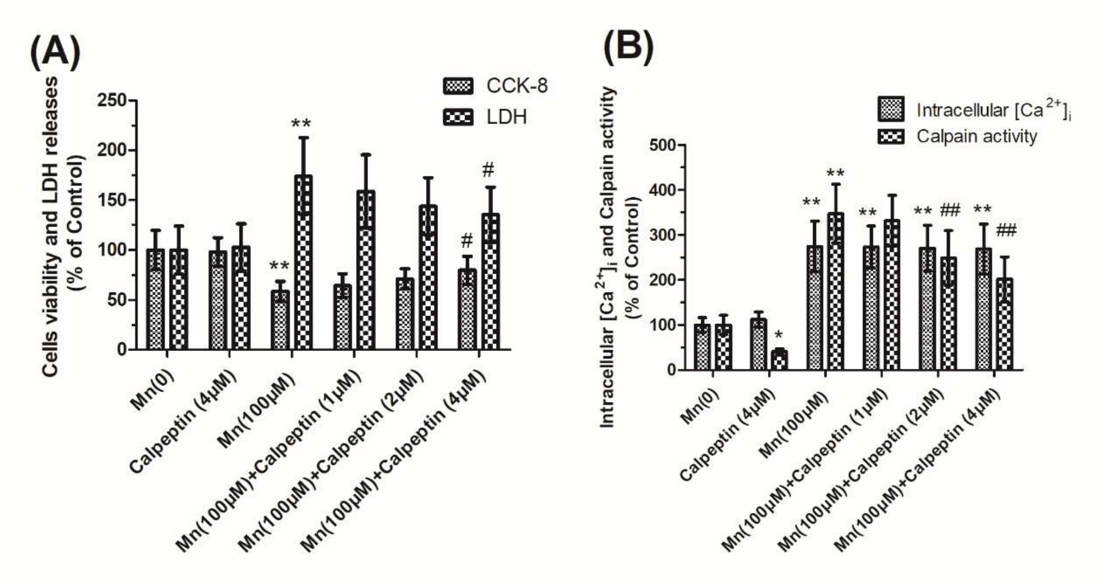


**Figure S2.** Calpeptin could ameliorate Mn-induced cell injury and the over-activation of calpains. (**A**) Cells injury was detected with LDH and CCK-8 assays. In cells pretreated with 4 μM calpeptin group, LDH release were significantly decreased compared with the cells only treated with 100 μM Mn (22.05%, $P < 0.05$). Data showed that calpeptin reduced Mn-induced cytotoxicity, the same results were obtained for CCK-8. Data were mean ± S.D, n = 6. (**B**) $[Ca^{2+}]_i$ in cells was calibrated from the measured fluorescence signals. $[Ca^{2+}]_i$ was increased in 100 μM Mn group (2.74-fold of control, $p < 0.01$), but had no obvious difference in calpeptin-pretreated groups compared with 100 μM Mn-treated group. Calpains activity was undertaken measuring the values of absorbance. 100 μM Mn-treated group resulted in calpains activity

increase (3.47-fold of control, $p < 0.01$). However, calpains activity decreased by 59.55% in cells treated with 4 μM calpeptin alone compared with the absolute controls ($p < 0.05$). Data were mean ± S.D., n = 6, *$p < 0.05$ and **$p < 0.01$ significantly different from the controls; $^{\#}p$ <0.01 and $^{\#\#}p$ < 0.01 significantly different from 100 μM Mn-treated group.

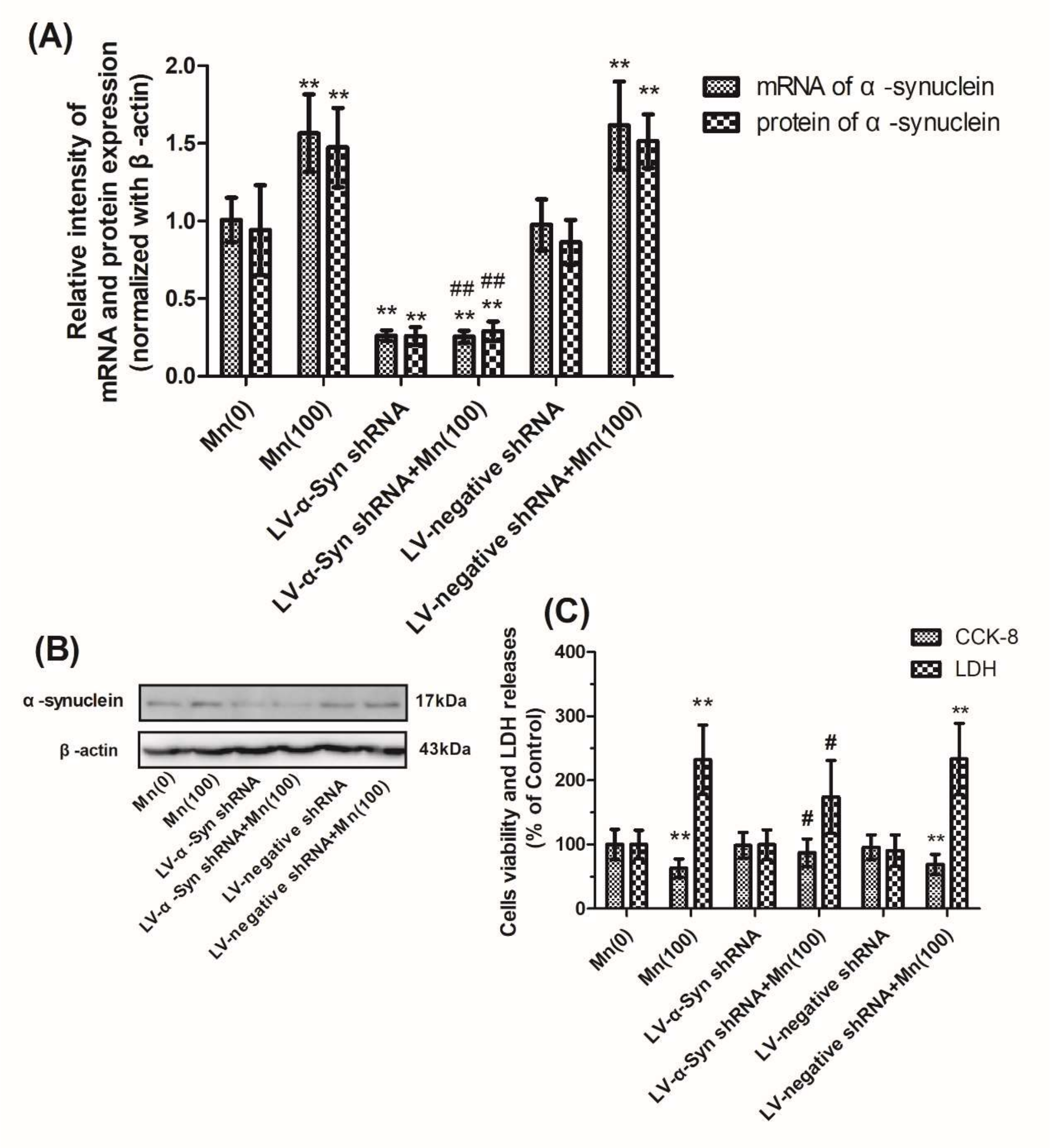


**Figure S3.** α-Syn shRNA reduced α-Syn expression level and ameliorated cell injury during Mn exposure. (**A**) The mRNA and protein expression of α-Syn in LV-α-Syn shRNA cells were significantly decreased (48.18% and 39.14% of the normal cells, respectively, $p < 0.01$), and there was no difference between the normal cells and LV-negative shRNA cells. Expression of α-Syn gene and protein were normalized with β-actin gene and protein respectively (n = 4). (**B**) The protein products of α-Syn and β-actin. (**C**) After the normal, LV-α-Syn shRNA and LV-negative shRNA cells were respectively exposed to 100 μM Mn for 24 h, LDH releases and CCK-8 were performed (n = 4). LDH release in 100 μM Mn-treated LV-α-Syn shRNA cells decrease significantly (24.91%, $p < 0.05$) compared with the normal cells. However, the 100 μM Mn-treated LV-negative shRNA cells decreased significantly (30.81%, $p < 0.01$) versus the

untreated normal cells. As it had in CCK-8 assay, 100 μM Mn-treated LV-$\alpha$-Syn shRNA cells had a significant increase (17.43-fold, $p < 0.05$) compared with the normal cells, the 100 μM Mn-treated LV-negative shRNA cells had a significant decrease (23.35%, $p < 0.01$) versus the untreated normal cells. Data were mean ± S.D, $^{*}p < 0.05$ significantly different from the controls; $^{\#}p < 0.05$ and $^{\#\#}p < 0.01$ significantly different from 100 μM Mn-treated group.

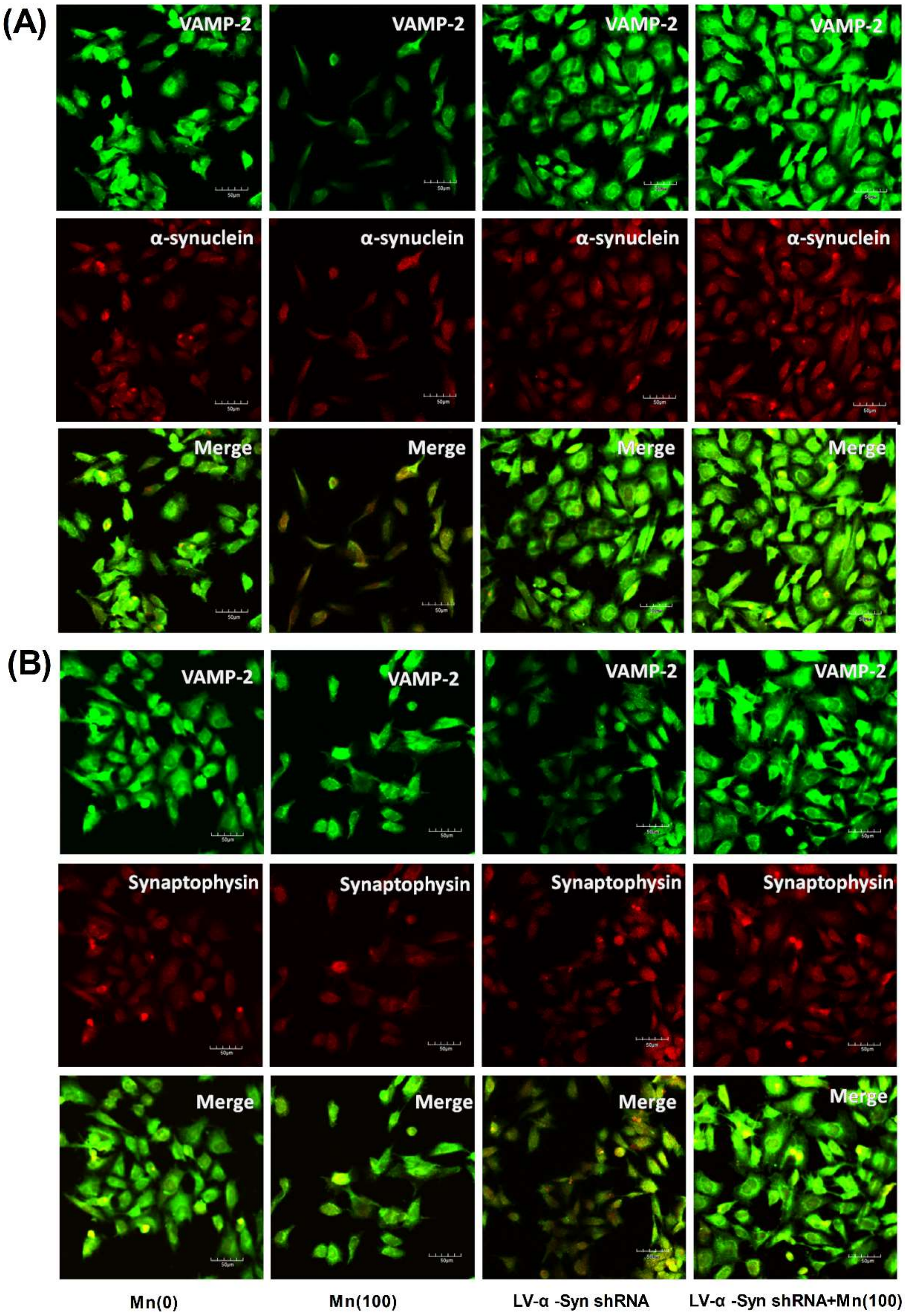

**Figure S4.** Co-localization fluorescence signals of VAMP-2 and $\alpha$-Syn, VAMP-2 and synaptophysin in cells. (**A**) Representative colocalization of VAMP-2 (green) and $\alpha$-Syn (red) in different groups. (**B**) Representative colocalization of VAMP-2 (green) and synaptophysin (red) in different groups. Scale bars: 50 μm.
